# Adaptive matching between phyllosphere bacteria and their tree hosts in a neotropical forest

**DOI:** 10.1186/s40168-020-00844-7

**Published:** 2020-05-21

**Authors:** Geneviève Lajoie, Rémi Maglione, Steven W. Kembel

**Affiliations:** Département des sciences biologiques, Université du Québec à Montréal, 141, Avenue du Président-Kennedy, Montréal, Quebec, H2X 1Y4 Canada

**Keywords:** Microbial communities, Phyllosphere, Functional traits, Host–symbiont matching, Metagenomic shotgun sequencing

## Abstract

**Background:**

The phyllosphere is an important microbial habitat, but our understanding of how plant hosts drive the composition of their associated leaf microbial communities and whether taxonomic associations between plants and phyllosphere microbes represent adaptive matching remains limited. In this study, we quantify bacterial functional diversity in the phyllosphere of 17 tree species in a diverse neotropical forest using metagenomic shotgun sequencing. We ask how hosts drive the functional composition of phyllosphere communities and their turnover across tree species, using host functional traits and phylogeny.

**Results:**

Neotropical tree phyllosphere communities are dominated by functions related to the metabolism of carbohydrates, amino acids, and energy acquisition, along with environmental signalling pathways involved in membrane transport. While most functional variation was observed within communities, there is non-random assembly of microbial functions across host species possessing different leaf traits. Metabolic functions related to biosynthesis and degradation of secondary compounds, along with signal transduction and cell–cell adhesion, were particularly important in driving the match between microbial functions and host traits. These microbial functions were also evolutionarily conserved across the host phylogeny.

**Conclusions:**

Functional profiling based on metagenomic shotgun sequencing offers evidence for the presence of a core functional microbiota across phyllosphere communities of neotropical trees. While functional turnover across phyllosphere communities is relatively small, the association between microbial functions and leaf trait gradients among host species supports a significant role for plant hosts as selective filters on phyllosphere community assembly. This interpretation is supported by the presence of phylogenetic signal for the microbial traits driving inter-community variation across the host phylogeny. Taken together, our results suggest that there is adaptive matching between phyllosphere microbes and their plant hosts.

Video abstract.

## Background

The phyllosphere—the aerial surfaces of plants including leaves—is a widespread microbial habitat that hosts a diversity of microorganisms that play key roles in plant ecology and evolution [1]. Phyllosphere microbes play key roles in plant health [2, 3] and human health [4], and can influence ecosystem function [5]. At a broad taxonomic scale, phyllosphere bacterial communities are consistently dominated by taxa including Actinobacteria, Bacteroidetes, Firmicutes, and Proteobacteria [6], indicating that plants also influence the composition of their microbial partners. A key goal of phyllosphere microbial ecology research has been to identify the adaptive basis of such relationships between plants and associated microbes.

Comparative studies of the taxonomic composition of phyllosphere microbial communities across plant hosts have demonstrated the importance of host identity as a key driver of variation in phyllosphere microbial taxonomic diversity. At fine taxonomic scales, the composition of these communities varies predictably across host plant species [7–9] and across genotypes within host plant species [10, 11]. Plants and associated bacteria also show cophylogenetic associations, with clades of plants and bacteria consistently occurring together [9, 12, 13], suggesting close adaptive associations between plants and their phyllosphere microbes.

Determining whether plant–microbe associations in the phyllosphere have an adaptive basis will require establishing how both plant and microbial functions are related across a range of host species. Plant functional traits—measures of morphology and physiology that capture key axes of variation in plant life history and ecology [14]—have been targeted as a potential proxy for explaining microbial community turnover among plant species. These traits determine the potential for nutrient, metabolite, and secondary compound leaching from the plant, which should largely determine the quality of a leaf as a habitat for phyllosphere microbes [15]. In support of this hypothesis, plant functional traits such as leaf mass per area, leaf elemental composition, and growth rate are correlated with phyllosphere microbial community turnover both within [16] and among plant species [12, 17–20].

Several studies have reciprocally identified the broad-scale microbial functional categories and adaptations that epiphytic microbes possess for living on plants [e.g. [16–19]]. Functions including the biosynthesis of osmoprotectants such as trehalose and betaine and the production of extracellular polysaccharides are enriched in the phyllosphere and are thought to provide key adaptations to life on leaf surfaces by allowing microbes to attach to the leaf surface and by providing resistance to environmental stresses and plant defenses [21, 22]. The enrichment of rhodopsin genes in leaf bacterial communities exposed to high light also points to a role for those pigments in improving microbial fitness through higher energy acquisition on sun leaves [23]. However, studies of microbial functions in the phyllosphere have largely been based on comparison of one or a few host plant species. How microbial functions map onto variation in host plant functions in diverse natural communities thus remains largely unknown. As a result, it is not clear whether plant microbiota exhibit the pattern of taxonomic turnover but functional homogeneity across hosts that has been observed in some animal microbiota [24] or if a turnover in microbial functions can also be observed across functionally different tree species.

In this study, we quantified the functional repertoire of microbial communities on leaves of multiple tree species in a neotropical forest on Barro Colorado Island (Panama) using metagenomic shotgun sequencing. Sampling was performed in a 50-ha long-term plot of old-growth tropical forest within which ~300 tree species have been recorded, most of them evergreen [25]. We asked which microbial functions are abundant in the phyllosphere, and how these functions are linked to the taxonomy and functional traits of plant hosts. Our central hypothesis was that the plant–microbe taxonomic associations previously observed in this forest [12, 26] should be driven by adaptive matches between microbes and host plants, leading to several key predictions. First, we predicted that microbial functions should vary among host plant species and be correlated with the functional traits of the hosts. Second, we predicted that cophylogenetic associations between trees and microbes should lead to a phylogenetic signal in microbial functions present on different plant hosts. Third, we predicted that microbial functions present on leaves should be filtered by the host since conditions on the leaves of different host plants create a selection pressure on the functions of microbes able to persist on those leaves.

## Results

### Metagenomic shotgun sequencing characterization of phyllosphere microbial functions

**Fig. 1:**
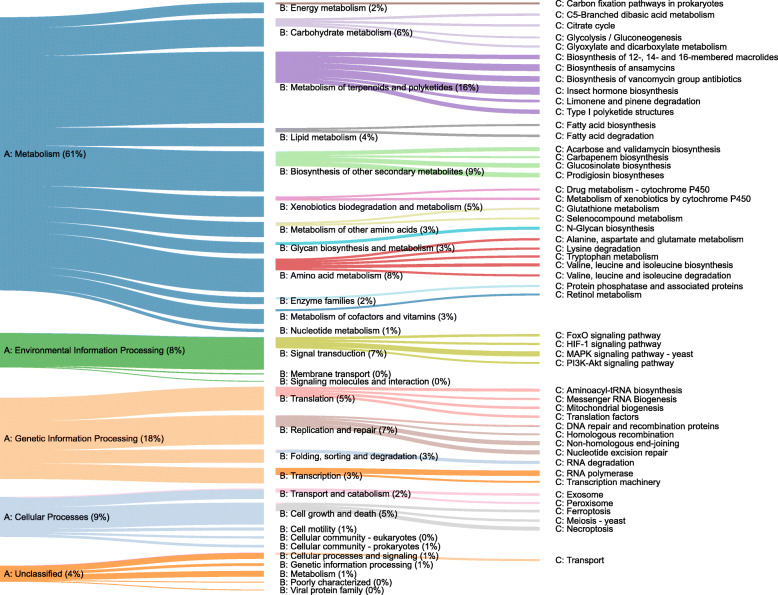
Relative abundance of the most abundant functional pathways detected across 24 tree phyllosphere samples in a neotropical forest in Panama. Functional pathways are classified using the KEGG functional hierarchy [27]

Overall, we detected 4587 different functional genes across all samples based on the annotation of metagenomic shotgun sequencing of tropical tree phyllosphere communities. Functions related to metabolism were the most abundant overall in our dataset, making up 45% of all functionally annotated sequences (Fig. 1). The principal metabolic functions in the phyllosphere were related to metabolism of amino acids (e.g., amino acid-related enzymes), nucleotides (e.g., purine and pyrimidine metabolism), carbohydrates (e.g., pyruvate, glyoxylate and dicarboxylate metabolism), and energy (e.g., oxidative phosphorylation & citric acid (TCA) cycle; Fig. 1). Groups of functional genes related to environmental and genetic information processing also had a high relative abundance, mainly membrane transport (e.g., transporters), translation (e.g., aminoacyl-tRNA biosynthesis), and signal transduction (e.g., two-component systems).

### Variation in phyllosphere functions and taxa among versus within samples

**Fig. 2:**
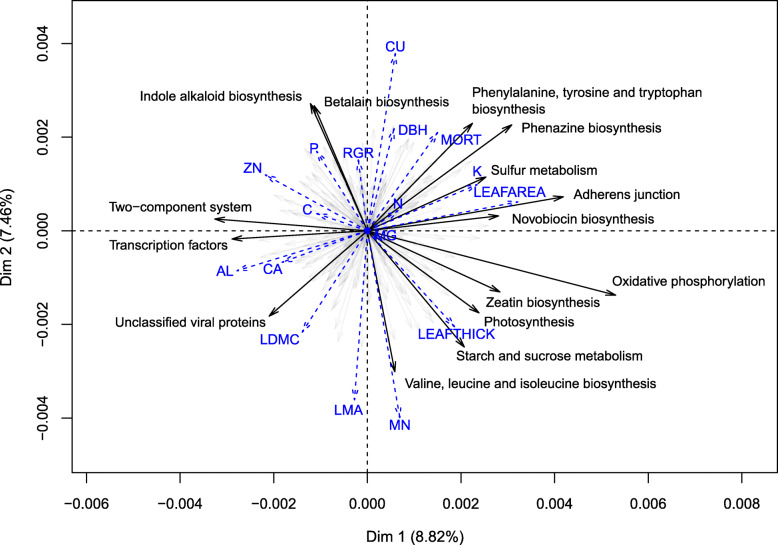
Principal components analysis (PCA) of microbial functional composition from the phyllosphere of neotropical trees. The 20 Tier 3 functions contributing the most to variation among samples are indicated as black arrow. Plant traits were fitted onto the PCA in a configuration that would maximize correlation with the PCA axes and are represented as blue dashed lines. Plant trait abbreviations are the following: aluminum (AL), calcium (CA), carbon (C), copper (CU), diameter at breast height (DBH), leaf area (LEAFAREA), leaf dry matter content (LDMC), leaf mass per area (LMA), leaf thickness (LEAFTHICK), manganese (MN), mortality (MORT), nitrogen (N), phosphorus (P), potassium (K), relative growth rate (RGR), and zinc (ZN)

**Fig. 3:**
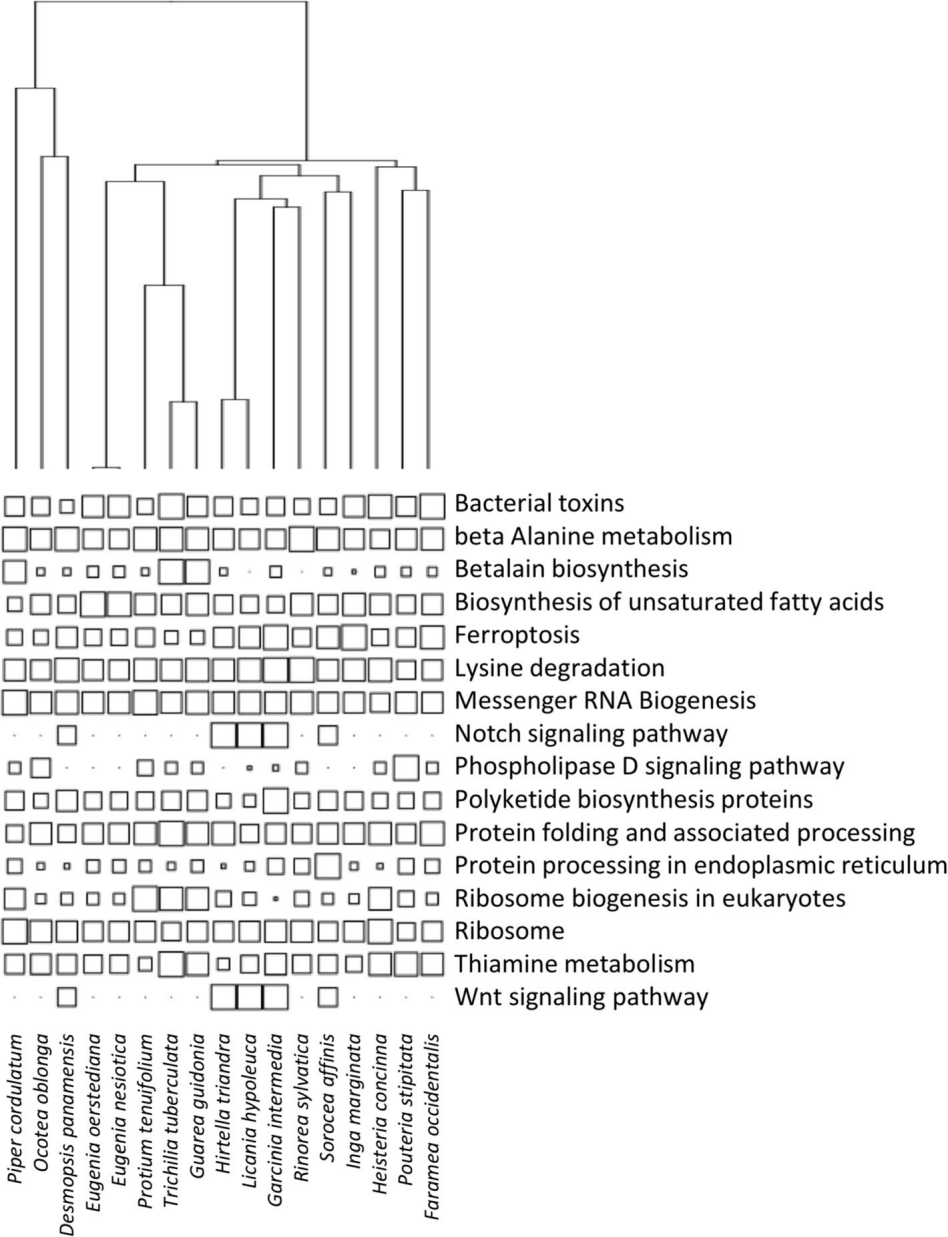
Distribution of microbial functions with respect to plant phylogeny. Distributions are shown for the subset of Tier 3 microbial functions with phylogenetic signal (K statistic) in the top 5% of values, as compared to the expected distribution of phylogenetic signal according to the K statistic randomization test (*P* < 0.05). Symbol size indicates the scaled relative abundance of microbial functions for each host species

The bacterial functions present on tree leaves were remarkably consistent among different samples. The vast majority of functional variation occurred within samples (> 97%), with a very small contribution of functional turnover among samples (< 3%) to total functional diversity, regardless of the functional level under study. Most taxonomic diversity was also observed within samples, with a contribution of beta-diversity increasing from 1 to 4.4% of the total diversity with a refinement of the taxonomic scale utilized (Table 1). The principal component analysis of bacterial community functional composition indicated that metabolic functions related to biosynthesis and degradation of secondary compounds and antibiotics as well as functions related to signal transduction and cell–cell adhesion were the most strongly varying among hosts (Fig. 2; Supp. Tab. 1, Additional File 1). We detected 16 Tier 3 functions that exhibited a strong phylogenetic signal with respect to the host phylogeny (functions with phylogenetic signal in the top 5% of values compared to null distribution compared to K statistic randomization test; *P* < 0.05; Fig. 3). These functions were mostly involved in the metabolism of terpenoids and polyketides, signal transduction, and cellular processes.

**Table 1:** Functional and taxonomic additive diversity partitioning of bacterial communities across 24 tree phyllosphere samples. The percentage of alpha diversity was calculated as the amount of alpha entropy divided by the amount of total entropy across all communities. The percentage of beta diversity was calculated as 1 minus the percentage of alpha diversity

	Functional			Taxonomic					
	Tier 2	Tier 3	Functional gene	Phylum	Class	Order	Family	Genus	Species
Alpha diversity (%)	100.0	99.8	97.2	99.0	99.0	99.0	98.8	98.2	95.6
Beta diversity (%)	0.0	0.2	2.8	1.0	1.0	1.0	1.2	1.8	4.4

### Associations between microbial and plant traits and host filtering

Many of the plant traits displayed some level of correlation with the principal axes of microbial functional community composition. Among these, morphological leaf traits (e.g., leaf area, leaf mass per area) were most strongly associated with the first two axes of microbial functional variation. Leaf elemental concentrations of copper, aluminum, and manganese were also strongly correlated with these first dimensions. The plant trait gradients explained altogether ~17% of variation in functional composition among microbial communities. The vast majority of the microbial Tier 3 functions were more or less abundant than 95% of the values obtained from the null model keeping both the total abundance of a trait and the number of traits in a community constant (Table 2). The filtering signal was similar for the microbial taxa and for the microbial functions (Table 2).

**Table 2:** Occurrences of Tier 3 functions and taxa across 24 tree phyllosphere samples. Occurrences of Tier 3 bacterial functions and taxa that are respectively more or less abundant than 95% of the values obtained from a null model randomizing abundances of functions and taxa across hosts (*n* = 9999)

	Number of combinations in the top 5% of the null model values	% of total	Number of combinations in the bottom 5% of the null model values	% of total	Total number of combinations
**Functions**					
Tier 3 functions	4360	70	930	15	6192
**Taxa**					
Phylum	1397	69	279	14	2016
Class	1073	63	405	24	1704
Order	2426	62	988	25	3888
Family	5597	64	2014	23	8808
Genus	26,723	66	6690	16	40,608
Species	183,337	76	23,479	10	240,288

## Discussion

The functional composition of tree phyllosphere microbial communities in a tropical forest in Panama is largely consistent with those reported in the literature, regardless of the type of plant studied, suggesting the presence of a core functional microbiota in phyllosphere microbial systems. Core functional microbiota in host-associated systems have also been reported for other hosts. Our study supports findings of an important role for the metabolism of carbohydrates and amino acids in bacterial survival in the phyllosphere [18, 28, 29] that is consistent with the abundance of these compounds in leaf leachates and photosynthates. The main mechanism of energy acquisition from these compounds appeared to be the TCA (citric acid) cycle, as reported in experimental studies of bacterial colonization of the phyllosphere [29]. Membrane transporters were also reported to be an important component of the epiphytic microbe functional repertoire, maximizing the ability to monopolize otherwise limiting resources [30]. The abundance of signal transduction functional pathways, involved in the rapid sensing and response to environmental change, would lastly be coherent with the high variability in conditions of humidity, light, and temperature in that microbial habitat [21].

The low functional variability in microbiota observed among tree species represents a further line of evidence supporting the presence of a core phyllosphere functional microbiota. This low variability, observed even at fine functional levels, could be the consequence of essentially similar constraints imposed by the generally harsh leaf environment on its microbial communities, regardless of the specific physiological traits of the host plant species. This low functional turnover among communities was also associated with a low taxonomic turnover, contrasting with reports from phyllosphere-associated temperate systems where species identity was a strong driver of taxonomic composition of the microbial communities [8]. These results could be explained by a finer-scale partitioning of taxa among neotropical rather than temperate tree species, or a greater overlap in species functional types limiting strong associations between microbial taxa and their hosts. Such differences should be further investigated.

Despite the high levels of convergence in microbial functions among the phyllospheres of different tree species, several lines of evidence support a role for plant species taxonomic and functional identity in driving microbial community assembly. Tree traits explained a notable portion of the functional turnover among microbial communities. Traits correlated with microbial functional turnover (e.g., leaf area, leaf mass per area) are mostly part of the leaf economics spectrum [31], a functional strategy scheme describing photosynthetic resource-use efficiency in plants, which is coherent with what we know of phyllosphere microbial physiology. The ability of a tree to be conservative of its resources and generate thicker and better protected leaves (i.e., high leaf mass per area) is likely to limit the leaching of nutrients from the leaf to the phyllosphere, in turn constituting a filter on resource-use strategies in microbes. The high correlation of leaf mass per area with turnover in microbial communities is coherent with a previously described role for cuticle characteristics in determining functional turnover among leaf microbial communities [16, 20]. The high correlation of aluminum and copper concentrations in leaves with microbial functional variation may be explained by their role as antibiotics. The predominance of two-component systems associated with high aluminum and copper concentrations suggests that the ability to sense and quickly respond to fluxes in these elements at the cell surface might constitute an efficient stress response to deal with these conditions [32]. This type of plant trait gradient is analogous to the leaf chemical gradient described by Yadav and colleagues [33], who reported variation in leaf colonization by phyllosphere microbes on different tree species as a function of their total leaf phenolics content. Taken together, these interpretations are concordant with the importance of energy metabolism, secondary metabolites, and antibiotic production as well as environmental sensing in driving functional turnover of microbes among tree species.

Other lines of evidence support the idea that the plant host plays a selective role on microbial community assembly, such as the detection of bacterial traits that exhibit a strong phylogenetic signal with respect to the host plant phylogeny. While this pattern might arise from the filtering of microbes on phylogenetically structured selective plant traits or from co-evolution of the two partners, it is regardless indicative of an influence of the host on the functional makeup of bacterial phyllosphere communities. Interestingly, the set of pathways that are important in driving functional turnover among communities belong to the same functional categories as the ones that are phylogenetically structured among plant hosts, supporting the proposed match between these bacterial functions and their host’s functional and taxonomic identity. The fact that the relative abundance of a large set of functions was different within communities than that expected by chance given their relative abundance across samples, also supports a role for individual tree species in structuring the functional composition of their phyllosphere bacterial communities. The higher filtering of most microbial taxa relative to microbial functions suggests a role for unmeasured trait variation in driving functional turnover among communities.

The relatively small but significant contribution of functional turnover among microbial communities to the total functional diversity observed across samples suggests that the functions that are of importance in driving the distribution of bacteria across different host trees are actually relatively few compared to those enabling the bacteria to pass the overall “phyllosphere filter” that is needed to survive in the phyllosphere habitat. It remains unknown whether the majority of functional pathways that do not vary among trees are actually important for the ecology of the microbes, or if that trait variation is adaptively neutral within communities. It is also possible that some pathways important for microbial adaptations to leaf physiological gradients are not yet functionally described and are part of the large number of sequences that could not be functionally annotated. Ongoing efforts to better characterize gene functions will help improve the precision of ecological inferences in environmental metagenomes.

## Conclusions

In conclusion, we have identified a core functional microbiota in the phyllosphere of neotropical trees. While most functional variation was observed within individual microbial communities, we reveal a functional matching between the traits of microbes and the traits of plants across 17 tree species, emphasizing the role for energy metabolism, secondary metabolites, and antibiotic production as well as environmental sensing in mediating bacterial adaptation to leaf trait gradients in the canopy. Our identification of the adaptive drivers of phyllosphere microbial community composition in this neotropical ecosystem represents a good starting point for identifying the types of microbial traits that could be routinely studied by phyllosphere microbial ecologists to address global questions on the ecological and evolutionary dynamics of phyllosphere microbes. Empirical testing of the fitness consequences of variation in those traits will represent an important next step in understanding adaptive processes in the phyllosphere.

## Methods

### Microbial DNA collection, extraction, and sequencing

Microbial communities were collected from the leaves of 24 individual trees from 17 tree species (1–2 samples per species) in the tropical lowland rainforest of Barro Colorado Island, Panama, in December 2010. These samples were selected from a larger pool of samples [12, 26] for which we had sufficient quantities of high-quality DNA, selecting host species to maximize the phylogenetic and functional diversity of hosts. Methodological details of sample collection are described by Kembel et al. [12]. Briefly, 50–100 g of fresh leaves were collected from the sub-canopy of one tree of each species. Microbial cells were then washed from each leaf sample using phosphate buffer [1 M Tris•HCl, 0.5 M Na EDTA, and 1.2% CTAB] and collected by centrifuging at 4000 × *g* for 20 min. DNA was extracted using MoBio PowerSoil DNA extraction kits and samples stored at − 80 °C for future analyses. We quantified DNA concentrations and sequenced both extraction negative controls and PCR negative controls for these samples as part of previously published analyses of bacterial 16S and fungal 28S amplicon sequencing of these samples [12, 26]; none of the negative control samples contained measurable concentrations of DNA, and upon sequencing, they contained fewer DNA sequences than the minimum cutoff for inclusion in analyses. As a result, they were all excluded from subsequent analyses in previously published studies and the present study. To quantify the metagenomic structure of each microbial community, we constructed a paired-end metagenomic shotgun library including a random sample of the whole community DNA composition using an Illumina Nextera XT® kit (Illumina reference FC-131-1024). These libraries were then sequenced using Illumina MiSeq paired-end 2 × 250 base pair sequencing (V2 kit, Illumina reference MS-102-2003). Analyses were performed on these 24 samples unless stated otherwise. Results were not influenced by including replicates of the same species (see tests below).

### Microbial taxonomy and functional trait annotation

Metagenomic shotgun sequencing yielded 14,642,408 reads in total. We trimmed sequences to remove Illumina adapters and truncate end-bases with a quality score less than 20, and removed sequences shorter than 25 bp, leaving 14,634,072 trimmed and quality-controlled reads. Taxonomic annotation of all sequences in each microbial community was performed to restrict functional analyses only to bacterial sequences. We annotated metagenomic reads using Kaiju, which annotates taxonomic identity of reads by comparing sequenced reads to the microbial subset of the NCBI BLAST non-redundant protein database [34]. Out of the 7,317,036 sequences, we were able to annotate taxonomy to at least the taxonomic level of domain/kingdom for 2,138,885 sequences, of which 2,100,491 sequences were from Bacteria, representing 29% of the total sequences. All subsequent taxonomic and functional analyses were based on this subset of sequences identified as belonging to the Bacteria. Of these Bacterial sequences, 1,902,749 were annotated to at least the phylum level, representing 26% of the total sequences. Analysis of taxonomic composition was carried out on this subset of sequences annotated to at least the bacterial phylum level. We rarefied all samples to 20,100 randomly chosen sequences per sample for taxonomic composition analyses, resulting in a total of 482,400 sequences for taxonomic analyses (relative abundances of major taxa are shown in Supp. Fig. 3, Additional File 2).

Functional annotation of microbial sequences was performed via protein homology searches using the KEGG annotation framework [27, 35] via the software COGNIZER [36]. Analyses resulted in the identification of functional genes and categories for 873,082 sequences representing 12% of sequences. In total, of the 7,317,036 bacterial sequences that were obtained from the metagenomic sequencing of all samples, 722,936 sequences were taxonomically annotated as bacteria and had a functional annotation. Only these sequences that were both bacterial and functionally annotated were used for the functional analyses. We lastly classified each of these sequences into functional categories, defined by the BRITE functional hierarchy manually curated for the KEGG annotation system based on published literature [27]. This hierarchy contains four different levels, which were designed as Tier 1, Tier 2, Tier 3, and functional genes, ranging from the more general to the more specific functional assignment (see [37]). Most analyses were performed at the Tier 3 level, in the intent of reaching a balance between the complexity of the data and its interpretability. In a few instances, Tier 3 categories were perfectly correlated across samples so we removed the duplicates from the dataset in order to reduce its dimensionality (Supp. Tab. 2, Additional File 1).

### Plant functional traits and phylogeny

We obtained measurements of plant functional traits for all plant species from a dataset collected previously on Barro Colorado Island [38]. This trait database initially included 21 whole-plant and leaf traits, but we reduced these traits to a subset of 16 traits with limited overlap in functional significance [39] (Supp. Fig. 2, Additional File 2). This reduced set of traits included height at maturity, sapling growth rate, and sapling mortality rate as whole-plant resource-use traits, leaf area and leaf dry matter content as leaf structural traits, and a suite of leaf elemental chemistry traits including concentration of aluminum, calcium, copper, magnesium, phosphorus, zinc, and nitrogen content. A phylogenetic hypothesis for host plant species was obtained by grafting tree species onto a dated megatree of angiosperms provided by Zanne et al. [40] using Phylomatic v.3 [41].

### Variation in phyllosphere functions among versus within samples

We determined the contributions of within- and among-sample variations in function of the total functional variation among metagenomic samples using additive diversity partitioning, where *γ*_div_ = *α*_div_ + *β*_div_ [42]. The percentage of alpha diversity was calculated as the amount of alpha entropy divided by the amount of total entropy across all communities. The percentage of beta diversity was calculated as 1 minus the percentage of alpha diversity. These metrics were calculated using the R package entropart [43]. Analyses were performed at three levels of functional aggregation (Tier 1 to Tier 3). We tested whether the presence of two samples rather than one for some of the sampled species would affect this diversity partitioning by subsampling the dataset to include all possible combinations of samples, totally a single sample per species (*n* = 128) and rerunning the analyses. This subsampling did not affect our results (Supp. Fig. 1, Additional File 2), such that we kept the 24 samples in the subsequent analyses. We then compared sources of turnover for functions and taxonomy between samples by performing the same analysis from the taxonomically annotated metagenomic sequences, defined at levels from phylum to species.

### Associations between microbial and plant traits

We performed a principal component analysis (PCA) of functional trait matrices and identified the functions contributing most to variation along the first axes of variation using R package FactoMineR [44]. We fitted the plant traits onto this ordination to identify correlations between bacterial traits driving the PCA and the plant traits. We evaluated the influence of tree species replicates in our samples on these results and did not uncover important differences in the main drivers of functional differences among samples when excluding these duplicates. We also performed a Procrustes analysis [45] on the duplicated samples to evaluate whether their functional composition were correlated and obtained a very high correlation coefficient (*r* = 0.989). As such, we can assume that individuals from the same species are structuring their leaf microbial communities in a similar way and do not drive important functional differences among samples. All 24 samples were thus kept in this analysis.

We quantified the phylogenetic signal in associations between microbial functions and host plant phylogeny using function *multiphylosignal* from R package Picante [46] to calculate Blomberg’s K. This statistic quantifies whether a microbial trait exhibits stronger phylogenetic signal than expected by chance under a Brownian motion model of trait evolution. The higher the K statistic, the more phylogenetic signal in the trait. We identified microbial functions with strong phylogenetic signal by comparing the variance of independence contrasts observed for each microbial function to those obtained through a null model where taxa labels have been shuffled across the tips of the phylogeny (*n* = 9999 randomizations). We considered a microbial function to exhibit strong phylogenetic signal if it fell in the top 5% of the distribution of signal based on the randomization test (*P* < 0.05 according to randomization test). We selected a single random sample per host species for those host species with more than one sample prior to calculating phylogenetic signal. We repeated this for different random subsamples and it did not qualitatively change the results so we report phylogenetic signal for a representative random subsample.

### Host filtering of microbial functions and taxa

The degree of host filtering on microbial communities was assessed by comparing the occurrence of traits in observed communities to those obtained from 9999 randomizations of community trait matrices. Host filtering was detected as an over- or under-representation of the given trait in individual communities. Randomizations were generated by permutations of the trait matrix preserving row and column totals. For each site and bacterial trait combination, we compare the observed frequency of the trait to the random values to assess whether it was lower or higher than expected by chance. To compare the strength of functional vs. taxonomic filtering, we applied the same procedure to the taxonomic datasets defined at each of six taxonomic levels, from the phylum to the species.

## Supplementary information

**Additional File 1.** Supplementary Tables. This additional file contains 2 supplementary tables, referred to in the main text.

**Additional File 2.** Supplementary Figures. This additional file contains 3 supplementary figures, referred to in the main text.

**Additional File 3.** Host phylogeny. A phylogenetic hypothesis for host plant species obtained by grafting tree species from the study site onto a dated megatree of angiosperms (see Methods for details).

## Data Availability

The datasets generated and/or analyzed during the current study are available in a MG-RAST repository: https://www.mg-rast.org/linkin.cgi?project=mgp91848. The scripts used to perform analyses for the current study are available in a GitHub repository: https://github.com/glajoie1/panama_metagenome.
