# Supplementary material for: Adaptive matching between phyllosphere bacteria and their tree hosts in a neotropical forest
Source: Microbiome. 2020 May 21;8:70. doi: 10.1186/s40168-020-00844-7 (PMC7243311; doi:10.1186/s40168-020-00844-7)
Supplement: Supplementary file 3 — Additional File 2. Supplementary Figures. This additional file contains 3 supplementary figures, referred to in the main text. [file 40168_2020_844_MOESM2_ESM.docx]

**Supplementary Figure Legends**

**Supp. Fig. 1.** Distribution of alpha, beta and gamma diversities generated from 128 subsampling of the metagenomic functional dataset to include only one sample per tree species. Despite variation observed among bootstraps, the relative importance of alpha vs. gamma diversity stayed constant at 97.3% alpha diversity and 2.7% beta-diversity for all subsamples. The red vertical line indicates the observed value.

**Supp. Fig. 2.** Distribution of trait values for 16 traits across 17 tree species from a neotropical forest. See caption of Figure 2 for a description of trait abbreviations.

**Supp. Fig. 3.** Relative abundance of phyla (A) and orders (B) across 24 leaf bacterial communities in a neotropical forest. Only the taxa that made up more than 1% of the total abundance of bacteria are indicated in the barchart.


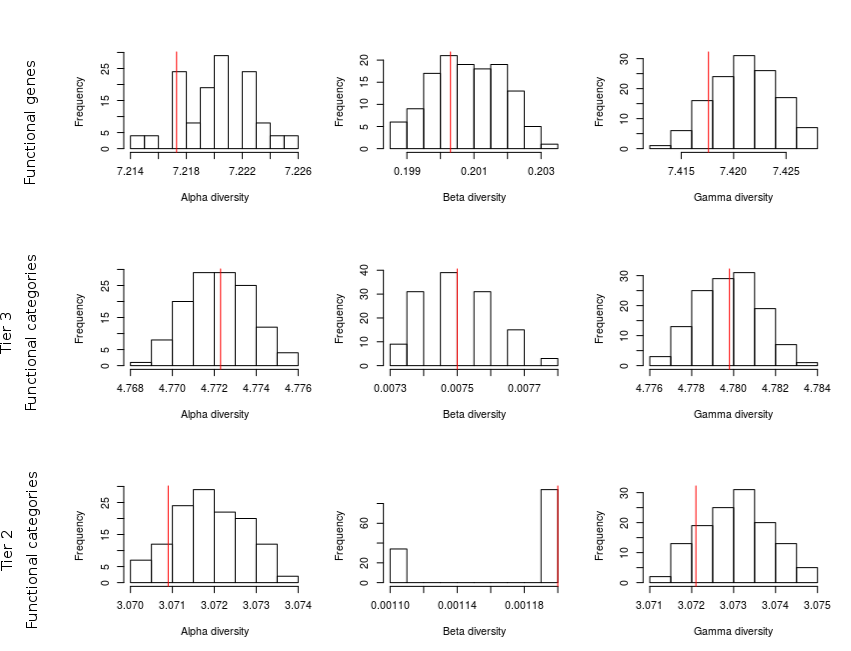


**Supp. Fig. 1.**


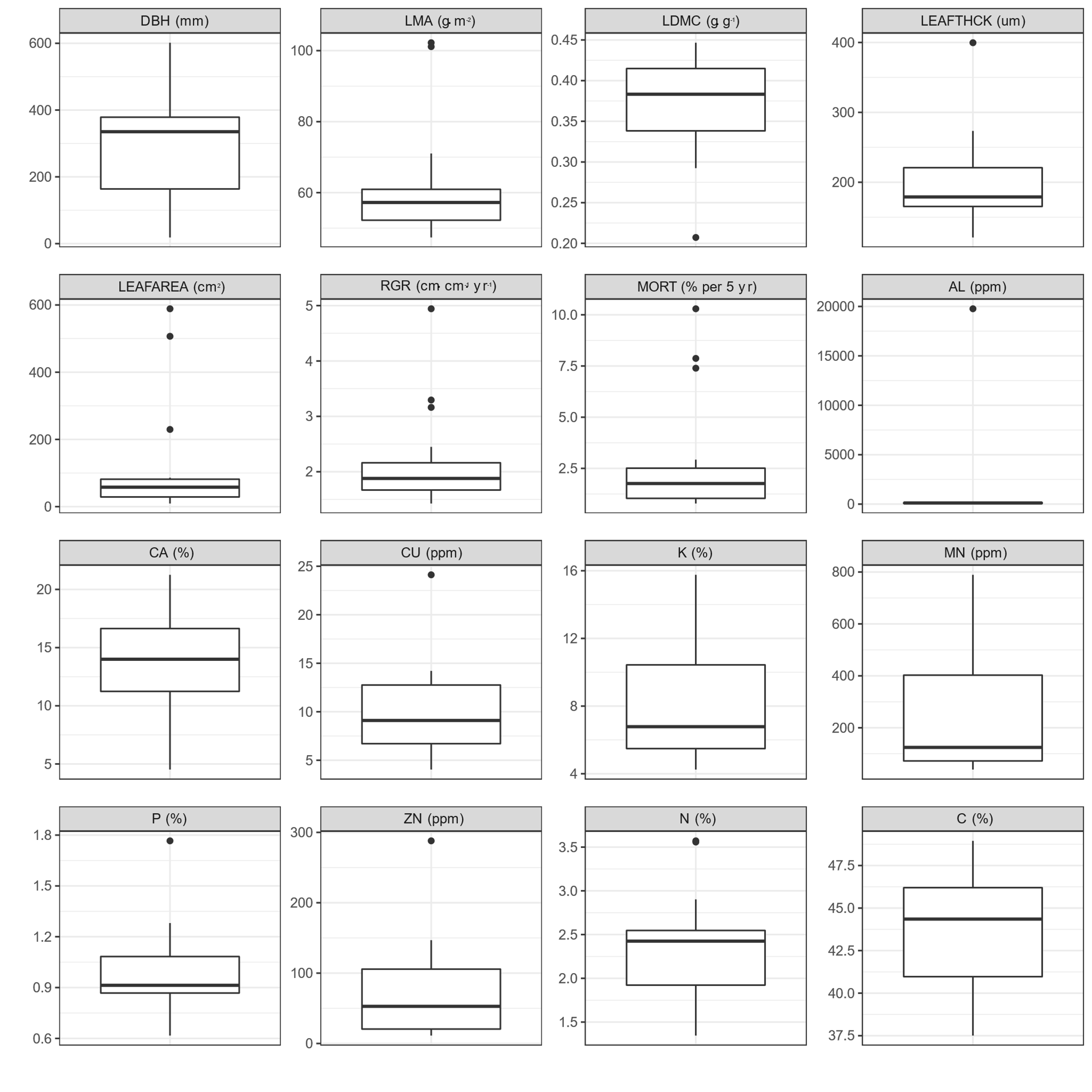


**Supp. Fig. 2.**


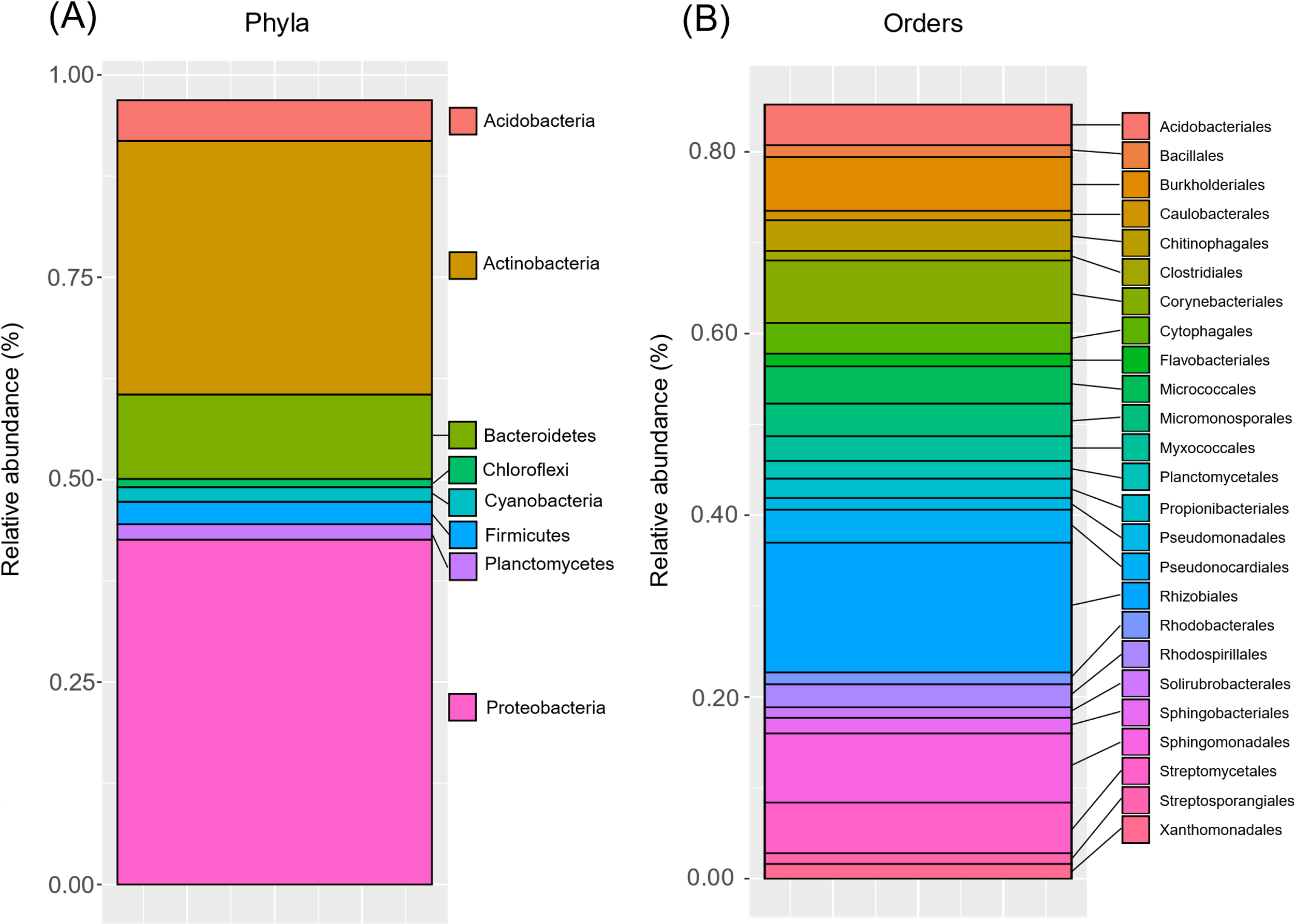


**Supp. Fig. 3**
